# Distribution of Native Lactic Acid Bacteria in Wineries of Queretaro, Mexico and Their Resistance to Wine-Like Conditions

**DOI:** 10.3389/fmicb.2016.01769

**Published:** 2016-11-08

**Authors:** Dalia E. Miranda-Castilleja, Ramón Álvar Martínez-Peniche, J. A. Aldrete-Tapia, Lourdes Soto-Muñoz, Montserrat H. Iturriaga, J. R. Pacheco-Aguilar, Sofía M. Arvizu-Medrano

**Affiliations:** Departamento de Investigación y Posgrado en Alimentos, Facultad de Química, Universidad Autónoma de QuerétaroSantiago de Querétaro, Mexico

**Keywords:** malolactic fermentation, multiplex PCR, *Oenococcus oeni*, starter cultures, wine spoilage

## Abstract

Native lactic acid bacteria (LAB) are capable of growing during winemaking, thereby strongly affecting wine quality. The species of LAB present in musts, wines during malolactic fermentation (MLF), and barrels/filters were investigated in wineries from the emerging wine region of Queretaro, México using multiplex PCR and culture. The resistance to wine-like conditions (WLC): ethanol (10, 12, and 13%), SO_2_ (30 mg⋅l^-1^), and low pH (3.5) of native LAB strains was also studied. Five species were detected within 61 samples obtained: *Oenococcus oeni*, *Lactobacillus plantarum*, *Pediococcus parvulus, Lactobacillus hilgardi*, and *Lactobacillus brevis*. Four species (excepting *L. brevis*) were found in must; *O. oeni* and *P. parvulus* were ubiquitous in wine and *L. plantarum* and *L. brevis* were mainly present at the initial stage of MLF, while *L. hilgardii* was mostly detected at the advanced stage. Furthermore, some species detected in barrel/filter, prove them to be hazardous reservoirs. From 822 LAB isolates, only 119 resisted WLC with 10% ethanol; the number of strains able to grow in WLC with 13% ethanol decreased approximately by 50%, *O. oeni* being the most versatile species with 65% of resistant isolates, while *Lactobacillus* spp. and *P. parvulus* were the most strongly affected, especially those recovered from barrel/filter, with less than 10% of resistant isolates. This study evidences the presence of local strains able to be used as starter cultures, and also enabled the assessment of the risks derived from the presence of spoilage LAB strains resistant to WLC.

## Introduction

The conversion from grape must into wine is a complex process that involves the development of various microorganisms, including lactic acid bacteria (LAB). However, wine is considered an unsuitable environment for microbial growth due to its low pH, high concentrations of ethanol and sulfur dioxide (SO_2_), and other limiting factors (Spano and Massa, 2006). The LAB capable of overcoming these conditions mainly belong to *Oenococcus*, *Lactobacillus*, *Pediococcus*, and *Leuconostoc* genera (Lonvaud-Funel, 1999).

In order to have any effect on wine quality, LAB should be able to not only survive, but also to grow within wine (Renouf et al., 2008), and the effect produced therein will depend on the major species present and their ability to overcome the harsh environment of winemaking (du Toit and Pretorius, 2000). The specie *Oenococcus oeni* is known as the main one responsible for malolactic fermentation (MLF), a process in which L-malic acid is decarboxylated into L-lactic acid, causing a partial deacidification, conferring microbial stability, and improving wine flavor profile (Lerm et al., 2010). However, some other LAB, such as *Pediococcus* spp. and some species of *Lactobacillus*, are widely associated with wine spoilage, often producing biogenic amines, off-odors, and other undesirable metabolites (Bartowsky, 2009).

Moreover, LAB can enter wine from vineyard or winery equipment (Fleet, 1993), and their diversity is influenced by grape variety and geographic region (Bokulich et al., 2013b). Therefore, it is advisable to study the autochthonous LAB of a particular winemaking area in order to detect potential starter cultures or species that represent risks of wine spoilage (Pérez-Martín et al., 2014). The use of molecular techniques to achieve this porpoise is currently preferred; some of them, such as ARDRA (Rodas et al., 2003), DGGE (Cocolin et al., 2013), or new generation sequencing (Bokulich et al., 2013a), display all the diversity of bacteria present in a sample. Meanwhile, other techniques, such as multiplex PCR described by Petri et al. (2013), are aimed at those bacteria of particular interest in winemaking. This particular technique allows the identification of 13 of the principal LAB associated with winemaking in a simple PCR assay, facilitating data processing or subsequent analyses to complete the identification of an amplicon.

Several studies intending to elucidate the presence, distribution, and adaptation of wine associated LAB have already been performed in wineries from regions with an extensive winemaking tradition, such as Mentrida (Pérez-Martín et al., 2014), La Rioja (González-Arenzana et al., 2015), Patagonia (La Hens et al., 2015), and Apulia (Garofalo et al., 2015). However, this kind of studies are missing in areas where the development of this industry is recent, like Queretaro State in Mexico. This region is considered nowadays the second most important within the Mexican territory. Located in the central area of the country, the climate is semi dry and temperate, the soils are deep with either a clayey loam texture or lightly calcareous. In 2013, above 350 ha of vineyards were censed and wine production was estimated in 1.5 millions of liters (Consejo Mexicano Vitivinícola A.C [CMV], 2014). To date, the main varieties established are ‘Merlot,’ ‘Cabernet Sauvignon,’ ‘Syrah,’ and ‘Tempranillo’ as well as the white varieties ‘Macabeo’ and ‘Chardonnay’ (Asociación de Vitivinicultores de Querétaro [AVQ], 2011). Wines possess low ethanol contents (from 9 to 12%) and a total titratable acidity around 7 g/L tartaric acid (De la Cruz-de Aquino et al., 2012). Wineries usually use commercial yeasts to guarantee an optimal alcoholic fermentation, but MLF is almost always carried out spontaneously, which makes it very unpredictable.

The aim of this research was to elucidate the principal LAB species present in strategic materials in wineries established in Queretaro and to determine their resistance to wine-like conditions (WLC), including high ethanol concentrations and low pH, in order to assess risks and detect possible starter cultures within local strains.

## Materials and Methods

### Experimental Site and Sampling

This study was conducted in four wineries named A, B, C, and D, located in Queretaro State, Mexico. Wineries A, B, and C have the respective vineyards and are located in the municipality of Ezequiel Montes, approximately 205 km from Mexico City. Winery D lacks a vineyard and is located 21 km from the others, in the municipality of Tequisquiapan. At winery C commercial cultures of LAB are used to induce MLF after finishing alcoholic fermentation; at winery B a commercial inoculum of LAB was used for the first time the year of the study, and at wineries A and D, MLF is left to occur spontaneously.

Depending on the availability at the wineries, different types of samples were collected, their characteristics are described in **Table 1**. Must, wine and barrel/filter samples were taken at winery A; must and wine at winery B; only must at winery C and only wine at winery D. Each type of sample was collected in triplicate as follows:

**Table 1:** Principal characteristics of the samples collected.

Winery	Sample type	N^1^	Sugar content (°Bx)	pH	Ethanol (%, v/v)	SO_2_ Total (mg⋅L^-1^)
A	Must	12	23	3.8	-	-
	Wine-i	3	5	3.4	12.1	31.5
	Wine-m	3	5	3.6	12.1	31.5
	Wine-a	3	5	3.7	12.1	31.5
	Barrel/filter	4	-	-	-	-
B	Must	15	22	3.8	-	-
	Wine-i	3	5	3.4	11.9	29.8
C	Must	9	21	3.7	-	-
D	Wine-i	3	4	4.1	12.6	33.1
	Wine-m	3	4	3.7	12.6	33.1
	Wine-a	3	4	3.8	12.6	33.1

^1^*Total number of samples.*

*Data reported as mean of three replicates per sample analyzed.*

(i) Must: Four mature bunches of grapes from the varieties: ‘Cabernet Sauvignon,’ ‘Tempranillo,’ and ‘Syrah’ at wineries A and B, and only ‘Macabeo’ at C, were randomly sampled in triplicates using plastic bags (20 cm × 30 cm). Also, 500 ml of must were taken from the stemmer of wineries A and B (one and two batches, respectively). Once they reached the laboratory, bunches were manually crushed inside their bags, the musts obtained from grapes and those collected from the stemmers were transferred to sterile flasks (500 ml) and left to spontaneously ferment at 25°C. For 15 days, aliquots of fermenting must were obtained every 5 days for molecular and microbial analyses.
(ii) Wine: Samples were taken once the alcoholic fermentation had ended. At wineries A and D, 100 ml of wine were sampled from three fermentation tanks, in three stages of MLF: (a) beginning, (b) intermediate, and (c) advance. At winery B, only the beginning stage was sampled, before a commercial strain inoculation. At each winery three types of wines were collected: two single-variety, one ‘Cabernet Sauvignon,’ another ‘Tempranillo,’ and the third a blend of ‘Grenache,’ ‘Carignan,’ ‘Syrah,’ and ‘Nebbiolo.’
(iii) Barrel/filter: The inside of a barrel was rinsed with 500 ml of peptone diluent (0.1%, pH 5), which was swirled five times; afterward the diluent was recovered in a sterile flask. Three filters were also individually collected in plastic bags. Once they reached the laboratory, 100 ml of peptone diluent was added to each filter and then homogenized in a Stomacher^®^ 400 (Seward Ltd.) at medium speed for 1 min.

### LAB Enumeration and Isolation

Must, wine and barrel/filter rinse aliquots (1 mL) were taken for serial dilutions and plated in three culture media: Man Rogosa Sharpe (MRS; DIBICO), MRS added to tomato juice (10%, v/v; Ruiz et al., 2008) or to apple juice (15%, v/v; Solieri et al., 2010). All media were adjusted to pH 4.8 and supplemented with natamycin (100 mg⋅l^-1^) and sodium azide (50 mg⋅l^-1^) to prevent yeast and acetic acid bacteria growth, respectively (Reguant et al., 2005). Incubation was carried out at 30°C for 8 days. As bacterial population is a non-normal data, the results were statistically analyzed using the non-parametric Kruskal – Wallis with Dunn’s *post hoc* test using the software JMP 9.0.

From culture plates, approximately 5% of the colonies were isolated and purified. Gram stain and catalase tests were performed to confirm the isolates belonging to LAB group. Isolates were preserved in MRS broth with glycerol 20% at -80°C until subsequent identification and resistance tests.

### Isolates Resistance to Wine-Like Conditions

The isolates’ ability to grow in the presence of ethanol, SO_2_, and low pH (WLC) was assessed through automatic readings of optical density (OD; every 20 min, for 72 h, at 30°C) using a Bioscreen^©^ analyzer (Miranda-Castilleja et al., 2015). Approximately 5 Log CFU⋅ml^-1^ (OD = 0.2) of each LAB isolate were inoculated in individual wells containing 200 μL of synthetic medium similar to wine (SW, Carreté et al., 2002) added to 53 mg⋅l^-1^ of potassium metabisulfite (equivalent to 30 mg⋅l^-1^ SO_2_), pH 3.5, and ethanol (10, 12, and 13%). As positive control, the isolates were also inoculated in the SW medium (pH 4) without the inhibitors. Detection time (DT), an indirect measure of the lag phase, was used as a response variable, considering the strain to be resistant to each condition when its DT value was lower than the total incubation time (72 h).

### Detection of LAB Species in Wineries

The detection of species present in the wineries’ samples (must, wine, and barrel/filter) and the identification of LAB isolates capable of growing in WLC were both carried out using a multiplex PCR (Petri et al., 2013).

#### DNA Extraction

Must, wine, and barrel/filter rinse aliquots (15 mL) were centrifuged (5000 × *g*, 10 min). From a cell pellet, DNA was extracted using the commercial kit Powersoil (MoBio Laboratories, Inc.) and the bench bead-top homogenizer PowerLyzer (MoBio Laboratories, Inc.) at 4500 rpm for 4 min, following the manufacturer’s instructions.

DNA extraction of LAB isolates was performed as follows: The strains were grown in 1 ml of MRS broth at 30°C for 3 days. The cell pellet obtained through centrifugation (13000 × *g*, 2 min) was re-suspended in 300 μl of lysis buffer (200 mM Tris–HCl, pH 8.5, 250 mM NaCl, 25 mM EDTA, 0.5% w/v SDS) with powdered glass (0.2 g). The suspension was shaken in a PowerLyzer (MoBio) at 4500 rpm for 1 min. After centrifugation at 13000 × *g* for 5 min, 150 μl of 3 M sodium acetate (pH 5.2) was added to the supernatant, which was stored at -20°C for 30 min and then centrifuged (13 000 × *g*, 10 min). The supernatant was transferred to a new tube and nucleic acids were precipitated with 400 μl of isopropanol and then washed with ethanol (70%). Finally, the DNA was re-suspended in 25 μl of TE buffer (Soto-Muñoz et al., 2014).

#### Multiplex PCR

The multiplex PCR was done using Multiplex Mastermix (Qiagen) with 1 μL of sample DNA, following the procedure described by Petri et al. (2013) with some modifications: 95°C for 15 min for initial denaturation, six cycles consisting of 30 s at 94°C, annealing for 3 min beginning at 69°C with a reduction of 1°C each cycle and an elongation step of 1.5 min at 72°C; then 25 cycles of 30 s at 94°C, 3 min at 62°C, and 1.5 min at 72°C, followed by a final extension step of 10 min at 72°C. The primers used are listed in **Table 2**. The PCR products were analyzed by electrophoresis on 1.8% agarose gels with TBE buffer (90 V for 45 min). Gels were stained with ethidium bromide (0.5 μg⋅ml^-1^) and visualized with an EDAS 290 digital imaging system (Kodak). Trackit^TM^ 100 bp (Invitrogen) was used as the standard molecular weight marker.

**Table 2:** Primers used for the identification of lactic acid bacteria (LAB) by multiplex PCR.

Primer	Sequence	Target
*Primer mixture I*		
SCAR-OENI-F	GGTAGATTAACCCGCGACG	*O. oeni*
SCAR-OENI-R	GGAATCGGTAGCATCCTG	
SCAR-LBR-F	GGAAGATCAAGAATATCGGTG	*L. brevis*
SCAR-LBR-R	GCGTCTCTAATTCACTGAGC	
SCAR-LPL-F	GAAGATTTGCCCATCGGTG	*L. plantarum*
SCAR-LPL-R	CGTTTGATGGTAGCGTTGC	
SCAR-LEU-F	GTGGTCATGGGTCTTAGC	*Leuconostoc*
SCAR-LEU-R	GGATCAAGACTAGCCAATGG	*mesenteroides*
SCAR-WPA-F	GCTGATGAACCCATACCTC	*Weissella paramesenteroides*
SCAR-WPA-R	GACCTGATTCGCTCGTTG	
SCAR-PDA-F	GTCTAAACTGGTGGTTAAACG	*P. damnosus*
SCAR-PDA-R	ATCGCACCTGGTTCAATGC	
SCAR-PPA-F	GCATGAATCACTTTTCGCTC	*P. parvulus*
SCAR-PPA-R	CAAAGATTGTGACCCAGTTG	
*Primer mixture II*		
SCAR-LBU-F	CTATCTTTAACCGCATTGCCG	*L. buchneri*
SCAR-LBU-R	GACACGCTTCTCATGATTGTC	
SCAR-PAC-F	ATGATGGACAGACTCCCTG	*P. acidilactici*
SCAR-PAC-R	CGAGCTGCGTAGATATGTC	
SCAR-LBH-F	TTCCTTGGTAATGTGCTTGC	*L. hilgardii*
SCAR-LBH-R	AATGGCAATCGCAATGGACG	
SCAR-PIN-F	CTATCCTTACAATGTGCATCG	*P. inopinatus*
SCAR-PIN-R	TGGTGCGTCAGTAAATGTAAG	
SCAR-LCU-F	CCAGATCCATCAGAAGATACG	*L. curvatus*
SCAR-LCU-R	GCTAACTTACCACTAACGACC	
SCAR-PPE-F	GGGAACGGTTTTAGTTTTATACG	*P. pentosaceus*
SCAR-PPE-R	CTAAGAGCGGTGATGATAAG	

## Results

### Enumeration and Isolation of LAB in Different Samples and Stages of MLF

**FIGURE 1:**
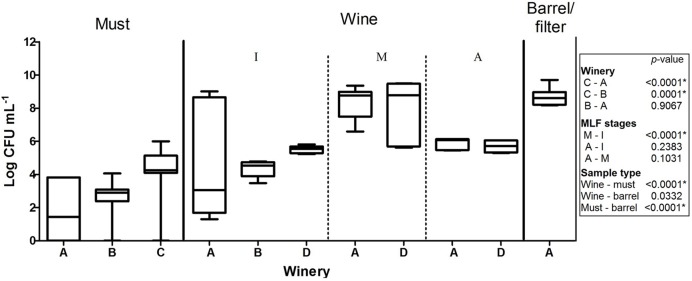
**Population of lactic acid bacteria in samples of must, barrel/filter and wine throughout three stages of malolactic fermentation: I beginning, M intermediate, A advanced; obtained from wineries (A–D).** Kruskal–Wallis analysis and Dunn’s *post hoc* test among wineries, MFL stages and sample type. The box indicates the 25th and 75th percentiles, line across the box shows the median value and the whiskers caps represent the maximum and minimum values.

A total of 822 isolates were recovered from the counting plates of the 61 samples collected at the four wineries (**Table 3**). Three culture media were used in this study to improve LAB recovery; however, contrary to previous reports (Solieri et al., 2010; Schillinger and Holzapfel, 2012), the population, the morphology of the colonies observed and species identified were very similar in the different media (**Supplementary Figure S1**). Therefore, in **Figure 1**, the LAB populations are shown, independent of culture media, involving six replicates of each sample analyzed (two per culture media). The LAB counts in musts from wineries A and B were rather low (10^1^–10^3^ CFU⋅ml^-1^) and no bacterial growth (<10 CFU⋅ml^-1^) was observed in several samples (5/12 in A and 6/15 in B). By contrast, higher counts (10^4^–10^5^ CFU⋅ml^-1^) were observed in musts from winery C, being this winery the one with the highest populations observed. In wine, the LAB populations ranged from 10^2^ CFU⋅ml^-1^ at the beginning of the process, to 10^9^ CFU⋅ml^-1^ at the second stage (climax of MLF), with intermediate values at the advanced stage. Finally, in barrel/filters rinse, the LAB population was around 10^8^ CFU⋅ml^-1^ being superior comparing to must but similar to the populations observed in wine.

**Table 3:** Number of samples handled and isolates obtained from the four wineries located in Queretaro, Mexico.

Winery	Sample type	Total samples	Total isolates
A	Must	12	23
	Wine	9	213
	Barrel/filter	4	156
B	Must	15	96
	Wine	3	89
C	Must	9	103
D	Wine	9	142
**Total**		**61**	**822**

### Detection and Distribution of LAB Species through the Wineries

Five species (*O. oeni*, *Pediococcus parvulus*, *Lactobacillus plantarum, Lactobacillus hilgardii*, and *Lactobacillus brevis*) were detected among the wineries’ samples (**Table 4**). In most of the cases, the detection by culture confirmed what was observed with the molecular detection (culture-independent). However, some discrepancies between detection approaches were observed: *L. brevis* in wines (from A and B) and barrel/filter was only detected by culture. Conversely, the presence of *O. oeni* at winery C was only determined by direct multiplex PCR.

**Table 4:** Percentage of incidence of LAB species detected by culture (C) and molecular assay (M) in samples of must, wine in three stages of malolactic fermentation (MLF): Initial (i), middle (m), and advanced (a) and barrel/filter; obtained in wineries A, B, C, and D.

Winery	Sample type	N^1^	*O. oeni*		*P. parvulus*		*L. plantarum*		*L. hilgardii*		*L. brevis*	
			M	C^2^	M	C	M	C	M	C	M	C
A	Must	12	0	0	0	0	58	17	0	0	0	0
	Wine-i	3	100	100	100	100	67	67	0	0	0	33
	Wine-m	3	100	100	100	100	0	0	0	0	0	0
	Wine-a	3	100	100	100	100	0	0	67	67	0	0
	Barrel/filter	4	100	100	100	100	0	50	100	100	0	50
B	Must	15	0	67	0	8	0	0	0	0	0	0
	Wine-i	3	100	100	100	100	0	100	0	0	0	100
C	Must	9	56	0	0	0	100	100	0	22	0	0
D	Wine-i	3	100	0	100	100	100	0	0	0	0	0
	Wine-m	3	100	33	100	100	67	0	0	0	0	0
	Wine-a	3	100	33	100	100	0	0	0	0	0	0

^1^*N: number of samples analyzed*.

^2^*Culture-dependent approach: identifying isolates resistant to wine-like conditions (WLC) with 10% ethanol.*

In several must samples (18/33), the LAB species investigated were not detected, and in the remaining ones, *L. plantarum* was widely detected at wineries A (58%) and C (100%). *O. oeni* was found in 67% of the samples from B and 56% from C. Finally, *P. parvulus* was only found in 8% of the samples from winery B and *L. hilgardii* only in 22% from C.

In wine samples, the five species were detected and *O. oeni* and *P. parvulus* were found in all samples. *L. plantarum* was detected in several samples from three wineries (22–56%). *L. hilgardii* was only found at winery A (22%), whereas, *L. brevis* was present at wineries A and B at 11 and 33%, respectively. Additionally, *L. brevis* and *L. plantarum* were mainly detected at the first stage of MLF, and *L. hilgardii* predominated at the advanced stage. Finally, in barrel/filter samples, all the five species were found. Winery A showed the greatest diversity of LAB species and at winery B the presence of *O. oeni* was remarkable.

### LAB Resistance to Increasing Ethanol Concentrations with SO_2_ and pH of 3.5

**FIGURE 2:**
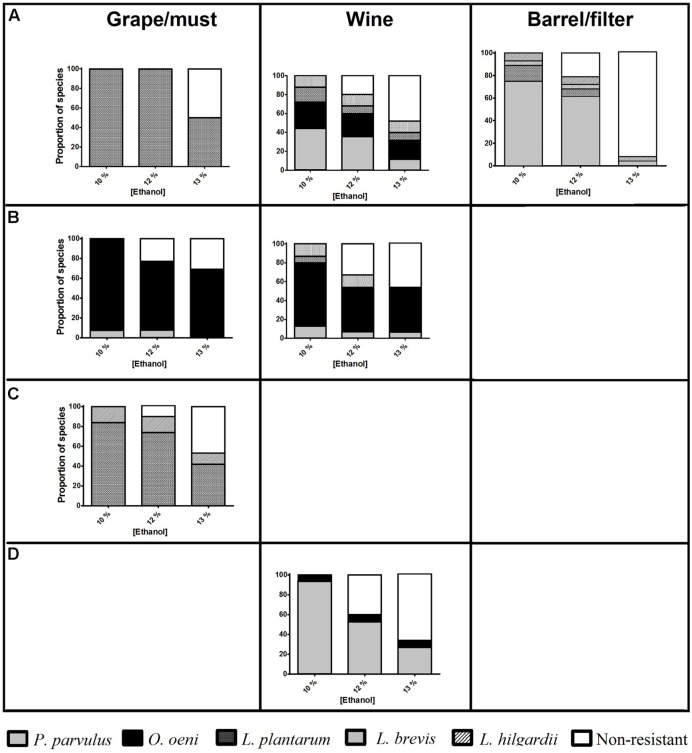
**Proportion of isolates of lactic acid bacteria (LAB) species capable of growing in a synthetic medium similar to wine (SW) with SO_2_ 30 mg l^-1^ pH 3.5 and different ethanol concentrations (% v/v, *x*-axis), from different wineries (horizontal boxes, A–D) and types of samples taken (vertical boxes).** The number of resistant isolates is indicated above the column.

As expected, the number of resistant isolates falls as ethanol concentration increases (**Figure 2**). In some samples (must from C; wine from A and D), the diversity of resistant species remained, with fewer individual ones capable of growing with 13% ethanol, evidencing strain variation. Moreover, the number of resistant *O. oeni* isolates remained unchanged, even with higher ethanol concentrations, which is particularly notable at winery B. Conversely, *P. parvulus* was strongly affected by higher ethanol levels, particularly those isolates obtained from barrel/filter, of which around 90% did not resist 13% ethanol. The *Lactobacillus* spp. in this study were also affected by 13% ethanol, with only 37% of the isolates being resistant to this condition. Finally, the high number of isolates (10 of 19) from must from winery C resistant to 13% ethanol is remarkable, given their origin.

## Discussion

### LAB Populations

The low LAB populations found in musts are consistent with the fact that they are minor constituents of grape microbiota, the populations usually reported being around 10^2^ CFU⋅g^-1^ (Barata et al., 2012). Meanwhile, higher populations found in must from winery C could be associated with grape variety; must from winery C was obtained from a white variety (‘Macabeo’), while musts from wineries A and B derived from red varieties (‘Tempranillo,’ ‘Syrah,’ and ‘Cabernet Sauvignon’). Higher numbers of LAB obtained from white varieties compared to red ones were also reported by Bae et al. (2006), which has been attributed to the fact that some phenolic compounds only present in red varieties can produce a toxic effect on bacteria (Reguant et al., 2000). The fluctuating populations of LAB observed in wine at different stages of MLF coincides with Saguir et al. (2009) and González-Arenzana et al. (2012), who reported that lower counts of LAB at the beginning of MLF increased throughout the process, reaching up to 8 Log CFU⋅ml^-1^.

Furthermore, barrel/filter samples were considered together in this study since the barrel contained the wine in which the filters were used, and only a few samples of each material could be collected. In particular, the LAB population found in barrels (10^3^ CFU⋅ml^-1^) was similar to that reported by González-Arenzana et al. (2013). Barrels are recognized as microbial reservoirs in cellars, since they offer shelter where microorganisms can remain. However, this material also represents a stressful environment, which could explain the low populations encountered therein (Renouf et al., 2007; Bokulich et al., 2013a).

### Detection and Distribution of LAB Species

**FIGURE 3:**
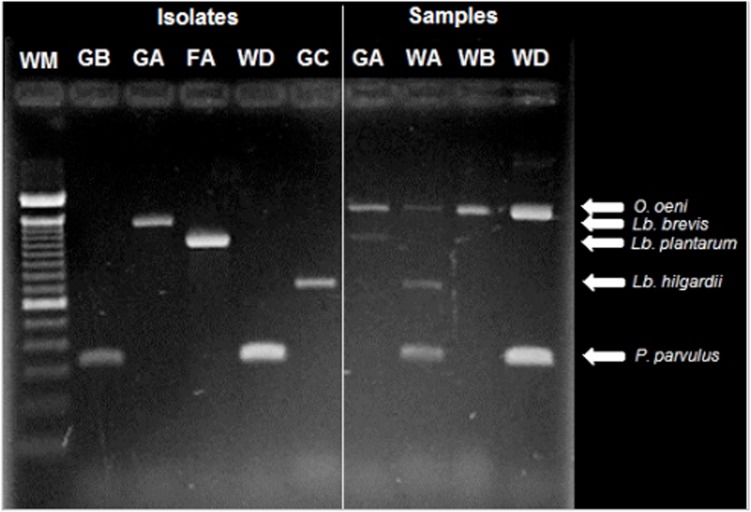
**Identification of LAB isolates (lines 2–5) and detection of species present in wine-related (6–9) samples using multiplex PCR.** The origin of the sample is indicated above as follows: The first letter corresponds to the type of sample: G, must; F, banel/filter; W, wine. The second letter corresponds to the winery: A, B, C, or D. The first line corresponds to the molecular weight marker Trackit^TM^ 100 bp (Invitrogen). Moreover, the species corresponding to that molecular weight is specified (Petri et al., 2013).

The multiplex PCR assay was efficient in detecting the principal LAB species in the winery samples (**Figure 3**), however, it was hampered when low LAB populations were present, as in musts and wines at the first stage of MLF. The detection limit reported for this technique is 10^4^ CFU⋅ml^-1^ (Petri et al., 2013), and the samples were concentrated 15 times, therefore, populations under 10^3^ were not detectable in this study. This detection limit could also explain the lack of recognition of *L. hilgardii, L. plantarum*, and *L. brevis* through this approach in some samples. Another known bias that could explain the lack of detection of certain species is preferential amplification, in which the abundance of certain species, such as *O. oeni* and *P. parvulus*, may have caused reagents to exhaust without amplifying scarce species (Sint et al., 2012).

The species mainly detected in musts (*L. plantarum, P. parvulus*, and *L. hilgardii)* are widely associated with wine grapes (Renouf et al., 2005; Bae et al., 2006; Barata et al., 2012). The last two are known to produce off-odors (Costello and Henschke, 2002) and biogenic amines in wine (Lonvaud-Funel, 2001), while *L. plantarum* has been recently regarded as starter culture for MLF (Lerm et al., 2011; Bravo-Ferrada et al., 2013), and has even shown additional advantages due to its capacity of degradation of biogenic amines (Capozzi et al., 2012) as well as better performance in co-inoculation with *Saccharomyces cerevisiae* (Berbegal et al., 2016). Moreover, the detection of *O. oeni* in musts is remarkable, given its importance in MLF and since this species is rarely found therein (Bae et al., 2006; Mesas et al., 2011).

In wine, the fact that *O. oeni* and *P. parvulus* were frequently found together suggests some type of association between them, as has been previously posited by Renouf et al. (2007) and Pérez-Martín et al. (2014). Nevertheless, it is important to point out that *P. parvulus* is the species most often involved in ropiness, a major bacterial alteration in wines (Dols-Lafargue et al., 2008). Moreover, the detection of *L. brevis* and *L. plantarum* only at the beginning of MLF shows a decrease in their populations at advanced stages, probably due to a low resistance to the modified medium. Finally, the fact that *L. hilgardii* was only found at the advanced stages of MLF suggests a contamination of the wine, probably through the barrels where this bacterium was also found; this emphasizes the need to implement effective disinfection methods during the winemaking process (González-Arenzana et al., 2013).

Even if the presence of some of these species can lead to wine spoilage, this problem has not been perceived in the local wines; only certain delays or inhibitions of the MLF are apparent. The spoilage features of these bacteria are usually strain-dependent, and for spoilage phenotypes to be produced, not only is the presence of the responsible bacteria required, but also the conducive environmental conditions, for instance, several stress conditions (ethanol, SO_2_, and low pH) promote the production of β-glucan responsible for ropiness by *P. parvulus* (Dols-Lafargue et al., 2008).

### Resistance to Wine-Like Conditions

In this study, LAB species were challenged with scarce nutrients combined with ethanol, SO_2_, and low pH, simulating a more realistic representation of what LAB face during winemaking. One of the principal changes in this process is ethanol concentration, which affects each LAB species differently, and the resistance of each isolate could also vary, depending on its origin (Arroyo-López et al., 2010).

The species showing more tolerant isolates to WLC was *O. oeni*, which is expected, since this species stands out for its ability to overcome the harsh conditions of wine, enabling it to dominate this media and establish itself in the cellars (Lonvaud-Funel, 1999). Conversely, higher ethanol levels significantly affected *P. parvulus*, an undesirable, but apparently prevalent species at these wineries. This high susceptibility could be due to the more stressful conditions found in barrels, which could lead to more sensitive strains.

Although *L. plantarum* has been previously reported with better adaptability to wine than *O. oeni* (G-Alegría et al., 2004), the isolates evaluated in this study did not show a remarkable performance. Even if *Lactobacillus* species are considered highly resistant to ethanol (Shane Gold et al., 1992), wines elaborated in Queretaro seldom reach more than 12% ethanol (De la Cruz-de Aquino et al., 2012), which could explain the lack of adaptation of local strains to 13% ethanol. Moreover, the fact that a high number of isolates (10 of 19) belonging to *Lactobacillus* spp. and obtained from winery C resisted 13% ethanol was surprising, since they were isolated from must, where they had not been previously exposed to alcohol. Winery C is the oldest one sampled (about 30 years old), which could have enabled some strains to adapt to both environments, vineyard and cellar. This allowed the identification of resistant strains that could eventually be used as starter cultures, as well as the detection of more hazardous species (and materials) with regard to spoilage.

## Conclusion

This is the first report related to the diversity of wine associated LAB in Mexico, and particularly in the wine region of Queretaro. Throughout the four wineries studied, five species (*O. oeni, P. parvulus, L. plantarum, L. hilgardii*, and *L. brevis*) were detected in must, wine, and barrel/filter samples. The species *O. oeni* and *L. plantarum* were detected at all the wineries and *P. parvulus* was only absent at winery C. *L. plantarum* and *L. brevis* were mainly found in musts and at the initial stages of MLF in wines, while *L. hilgardii* was principally detected at the end of MLF. The highest ethanol concentration tested (13%) combined with 30 mg⋅l^-1^ of SO_2_ and pH of 3.5 diminished the number of resistant isolates by around half, regardless of materials origin, with *O. oeni* being the species with a greater proportion of resistant isolates. In contrast, *P. parvulus* and *Lactobacillus* species obtained from barrel/filters were the most affected by high concentration of ethanol.

## Author Contributions

All the authors have revised the present draft, contributed important intellectual content and approved the final version to be published. They have also agreed to be accountable for the content of the work. DM-C: Conception of work, acquisition, analysis and interpretation of data, draft development. RM-P: Conception of work and experimental design, consultation on wine quality aspects. JA-T: Support with data acquisition, molecular techniques, and microbial diversity. MI: Consultation on microbial diversity and microbial kinetic studies. LS-M: Consultation on molecular techniques and data interpretation. JP-A: Consultation on sampling at wineries and microbial metabolism in vineyards. SA-M: Conception and design of work, expertise in lactic acid bacteria characterization, data analysis and interpretation, draft development, and financial support management.

## Conflict of Interest Statement

The authors declare that the research was conducted in the absence of any commercial or financial relationships that could be construed as a potential conflict of interest.

## Supplementary Material

FIGURE S1 **Proportion of LAB species recovered in three media from must, wine and barrel/filter rinse: MRS, Man Rogosa Sharpe; MRS-T, MRS added to tomato juice; and MRS-AJ, MRS added to apple juice**.
