# Supplementary figures and images for: Distribution of Native Lactic Acid Bacteria in Wineries of Queretaro, Mexico and Their Resistance to Wine-Like Conditions
Source: Front Microbiol. 2016 Nov 8;7:1769. doi: 10.3389/fmicb.2016.01769 (PMC5100547; doi:10.3389/fmicb.2016.01769)

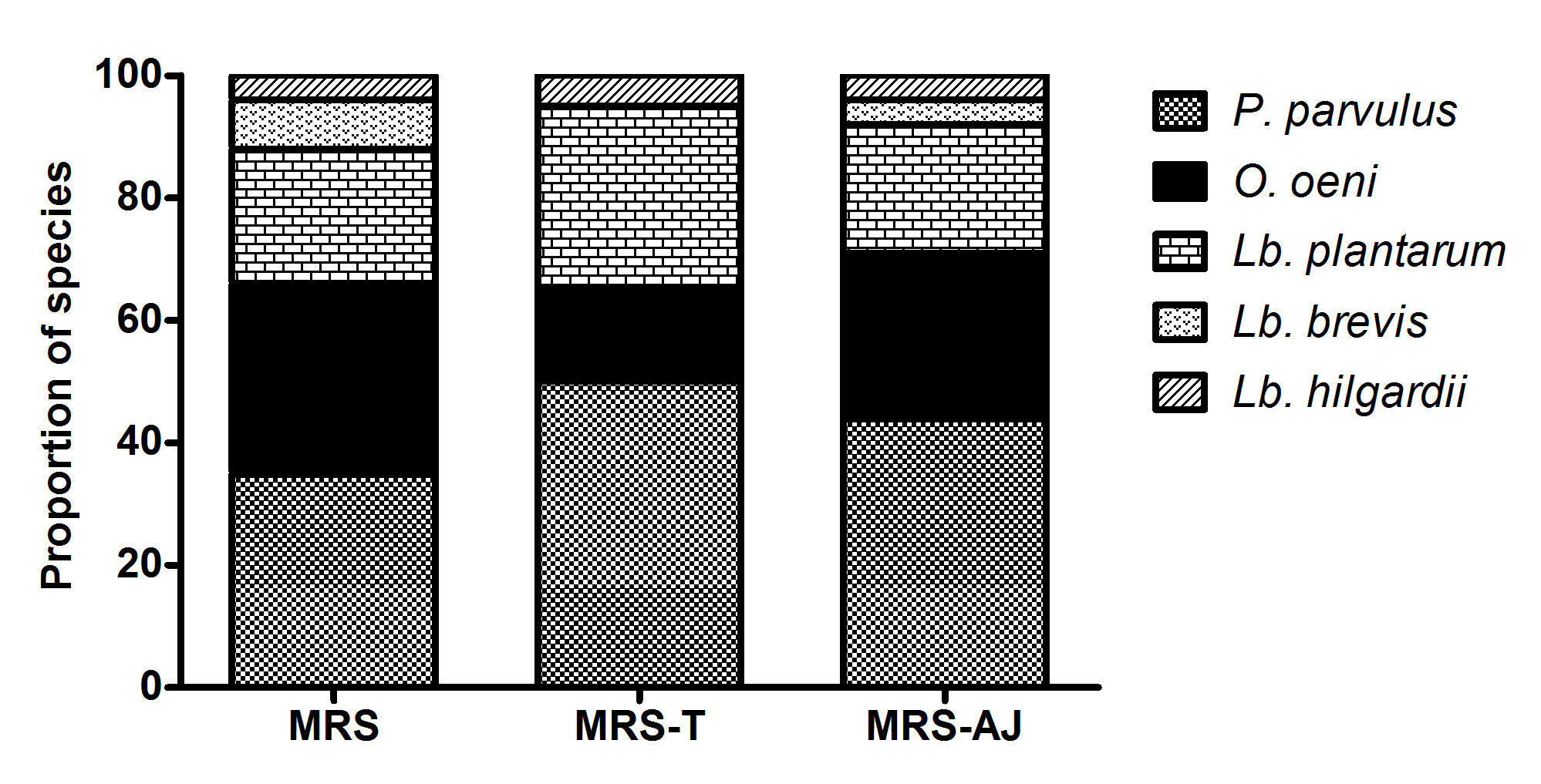

Supplement: FIGURE S1 — Proportion of LAB species recovered in three media from must, wine and barrel/filter rinse: MRS, Man Rogosa Sharpe; MRS-T, MRS added to tomato juice; and MRS-AJ, MRS added to apple juice. [file Image_1.JPEG]
